# Molecular Characterization of a Novel Integrative Conjugative Element ICE*Hpa1* in *Haemophilus parasuis*

**DOI:** 10.3389/fmicb.2020.01884

**Published:** 2020-08-13

**Authors:** Hua-Run Sun, Xiao-Die Cui, Xiao-Kang Liu, Shuai-Hua Li, Kai-Fang Yi, Yu-Shan Pan, Hua Wu, Li Yuan, Gong-Zheng Hu, Dan-Dan He

**Affiliations:** Department of Pharmacology and Toxicology, College of Animal Science and Veterinary Medicine, Henan Agricultural University, Zhengzhou, China

**Keywords:** multiresistance, *H. parasuis*, ICE, conjugation, transposon

## Abstract

ICE*Hpa1* was identified in the genome of a serovar 8 *Haemophilus parasuis* ST288 isolate YHP170504 from a case of swine lower respiratory tract infection. The aim of the present study was to characterize the integrative conjugative element ICE*Hpa1* and its multiresistance region. Susceptibility testing was determined by broth microdilution and the complete ICE*Hpa1* was identified by WGS analysis. The full sequence of ICE*Hpa1* was analyzed with bioinformatic tools. The presence of ICE*Hpa1*, its circular intermediate and integration site were confirmed by PCR and sequence analysis. Transfer of ICE*Hpa1* was confirmed by conjugation. ICE*Hpa1* has a size of 68,922 bp with 37.42% GC content and harbors 81 genes responsible for replication and stabilization, transfer, integration, and accessory functions, as well as seven different resistance genes [*bla*_Rob–__3_, *tet*(B), *aphA1*, *strA*, *strB*, *aac(6)′-Ie-aph(2′)-Ia*, and *sul2*]. Conjugation experiments showed that ICE*Hpa1* could be transferred to *H. parasuis* V43 with frequencies of 6.1 × 10^–6^. This is the first time a multidrug-resistance ICE has been reported in *H. parasuis*. Seven different resistance genes were located on a novel integrative conjugative element ICE*Hpa1*, which suggests that the ICE*Hpa1* is capable of acquiring foreign genes and serving as a carrier for various resistance genes.

## Introduction

The gram-negative bacterium *Haemophilus parasuis* is the causative agent of Glasser’s disease characterized by polyarthritis, fibrinous polyserositis, and meningitis in swine (Oliveira and Pijoan, 2004). The *H. parasuis* infection may cause great economic losses to the global pig industry (Oliveira et al., 2001).

More and more attention has been drawn to the antimicrobial resistance in bacteria from food-producing animals. In *H. parasuis*, the resistant genes are usually located on small plasmids, in which *mob* genes (*mobA*, *mobB*, *mobC*, *mobA-like*, *mobC-like*, and *mobA-L*) and IS*Apl1* are usually identified flanking the resistant genes (Lancashire et al., 2005; Chen et al., 2010; Yang et al., 2013; Li et al., 2015; Moleres et al., 2015). However, no other mobile genetic elements [transposons, integrons, and integrative and conjugative elements (ICEs)] have been found to be associated with the resistant genes in *H. parasuis*. ICEs are self-transmissible mobile elements that are widespread among different bacteria (Burrus et al., 2002; Burrus and Waldor, 2004; Lei et al., 2016). ICEs are composed of a set of core genes that are responsible for replication, maintenance, conjugation, recombination, and regulation, with other accessory modules, such as antimicrobial resistance genes (Robinson et al., 2013; Johnson and Grossman, 2015). In addition, ICEs usually have a single insertion site, which is often in the 5′ or 3′ end of a tRNA or other highly conserved genes such as the gene *prfC*, in the chromosome of their host (Mulvey et al., 2001; Johnson and Grossman, 2015). ICEs, as vehicles for active DNA exchange among different bacteria, contain some specific genes or sites needed for processing their DNA for transfer. Most of these genes are not expressed when the ICE is integrated in the chromosome; however, expression of the genes needed for excision, integration, and conjugation is induced under certain conditions, and the ICE may excise from the host chromosome to form a dsDNA circular intermediate. Some ICE-encoded proteins assemble into a mating pore that is responsible for transferring the ICE. The new host may recognize the origin of transfer (oriT), process the ICE dsDNA to generate a linear ssDNA-protein (T-DNA) through the ICE-encoded relaxase, and pump the T-DNA into the recipient. Then the ICE was recombined into the new host chromosome through an ICE-encoded integrase (Toleman and Walsh, 2011; Johnson and Grossman, 2015; Wright et al., 2015).

Quite a few ICEs have been identified in *Pasteurellaceae*, such as *Haemophilus influenzae*, *Pasteurella multocida*, *Mannheimia haemolytica*, and *Actinobacillus pleuropneumoniae* (Juhas et al., 2007; Brenner et al., 2012; Eidam et al., 2015; Bossé et al., 2016; Li Y. et al., 2018). However, no complete multidrug-resistance ICE in *H. parasuis* has been described in detail to date. In this study, we identified ICE*Hpa1*, a novel ICE carrying multiple resistance genes, in the chromosome of a serovar 8 *H. parasuis* ST288 isolate YHP170504, in a feedlot from Henan, China, in 2017.

## Materials and Methods

### Bacterial Strains and Susceptibility Testing

The strain YHP170504 was obtained from a case of swine lower respiratory tract infection in a feedlot from Henan, China, in 2017. Owing to the unavailability of an approved method for *H. parasuis*, MICs of *H. parasuis* isolates were determined using broth microdilution method following CLSI standard (Clinical and Laboratory Standards Institute [CLSI], 2018) for *A. pleuropneumoniae*. The antimicrobial agents tested were oxytetracycline, doxycycline, ampicillin, amoxicillin, ceftiofur, cefquinome, enrofloxacin, streptomycin, gentamicin, tilmicosin, tylosin, florfenicol, sulfamethoxazole/trimethoprim (19/1), lincomycin, and colistin. *A. pleuropneumoniae* ATCC27090 and *Escherichia coli* ATCC 25922 were used as control strains.

### WGS and Analysis

Total genomic DNA of strain YHP170504 was extracted using the TIANamp Bacteria DNA Kit (TIANGEN, Beijing, China) and subjected to WGS using Illumina Nextseq 500 and the Oxford Nanopore Technologies (ONT) MinION platforms. Sequencing reads including short-read and long-read data were assembled using Unicycler 0.4.4 with the hybrid strategy (Wick et al., 2017; Li R. et al., 2018). The complete sequence of ICE*Hpa1* was initially annotated using the RAST server^1^ and corrected manually. Comparison analysis was conducted using the genome comparison visualizer Easyfig.

### Confirmation of the Circular Extrachromosomal Form of the ICE and Conjugal Transfer of ICE*Hpa1*

Chromosomal insertion sites were confirmed by PCR in YHP170504 and transconjugants. For the 5′ junction, primers F1 and R1 were designed to amplify a 515-bp fragment from upstream of the ICE*Hpa1* insertion to a sequence within the 5′ end of ICE*Hpa1*. For the 3′ insertion, primers F2 and R2 were designed to amplify a 458 bp fragment from within the 3′ end of ICE*Hpa1* insertion to a sequence of the downstream of the ICE*Hpa1* insertion. To confirm the extrachromosomal circular form of the ICE, outward-facing primers ICE-out-F and ICE-out-R were used.

To investigate self-transfer ability of the ICE*Hpa1*, the conjugation assay was performed using the *H. parasuis* strain YHP170504 as the donor and *H. parasuis* V43 (rifampicin resistance) as the recipient. The serovar 4 *H. parasuis* ST170 isolate V43 was from the strain collection of our laboratory, and the rifampicin-resistant mutant of this strain was generated by selection on Tryptic Soy Agar (TSA) plates supplemented with 10% fetal bovine serum, 10 mg/L nicotinamide adenine dinucleotide (NAD), and increasing rifampicin concentration. For the conjugation assay, overnight cultures of donor and recipient strains grown in Tryptic Soy Broth supplemented with 10% fetal bovine serum and 10 mg/L NAD were mixed (1:5) and incubated for 4 h at 37°C. Bacterial cultures were spread on TSA plates supplemented with 10% fetal bovine serum, 10 mg/L NAD, oxytetracycline (8 mg/L), and rifampicin (100 mg/L), incubated at 37°C for 24 h. Then the transconjugants were screened on the plates. The conjugation frequency was calculated as the number of transconjugants per donor. All the transconjugants were confirmed with PCR using the primers *virB4*-F and *virB4*-R, susceptibility testing, and MLST. All the primers used are listed in Table 1.

**TABLE 1:** PCR primers used in this study.

Target	Primer	Sequence 5′–3′	Amplicon size (bp)	Annealing temperature (°C)
5′ junction of ICE*Hpa1*	F1	ATGTGGTGAATATTTAACTA	515	53
	R1	GGTGCAGAATCTTCAATATG		
3′ junction of ICE*Hpa1*	F2	TCTAGACTTTACAAGAAAAC	458	50
	R2	AGCTACATTGACTATAACGC		
Circular form and insertion	ICE-out-F	CGAGTGAAAAATTCATACAA	712	47
sites of the ICE*Hpa1*	ICE-out-R	TGGTTTCCCTATTTCTAGCC		
*virB4*	*virB4*-F	CAATACAAGCCATCGCTATC	393	55
	*virB4*-R	TTTGTCTTCGAATAGACCAC		
Circular form of the Tn*6742*	Tn-out-F	GGCTATTTCACCCACGCACT	1036	55
	Tn-out-R	TGAGTACTTCCTACCGACAT		

### Serotyping and Multilocus Sequence Typing (MLST)

The strain YHP170504, V43, and the transconjugant V43::ICE*Hpal1* were typed by serotyping and MLST. Serovars of the strains were determined using the primers Howell previously described (Howell et al., 2015). Seven housekeeping genes (*atpD*, *infB*, *mdh*, *rpoB*, *6pgd*, *g3pd*, and *frdB*) were amplified and sequenced as described previously (Mullins et al., 2013), after registration of sequences at https://pubmlst.org/hparasuis/ for assignment of allele numbers and STs; data were analyzed using software available on the website.

### Evolutionary Analyses of the Integrase

The integrase, an important core gene of ICE, is needed for both integration and excision. Evolutionary analyses were conducted in MEGA7 (Kumar et al., 2016) and the analysis involved 11 integrases complete sequences of eight ICEs from *Pasteurellaceae*. Initial trees for the heuristic search were obtained automatically by applying Neighbor-Join and BioNJ algorithms to a matrix of pairwise distances estimated using the maximum composite likelihood approach and then selecting the topology with superior log-likelihood value (Kumar et al., 2016).

### Nucleotide Sequence Accession Number

The complete sequences of the chromosome and the ICE*Hpa1* in *H. parasuis* YHP170504 have been submitted to GenBank with the following accession numbers: CP054198 and MN844034.

## Results and Discussion

The strain YHP170504 was serotyped as serovar 8, belonging to ST288. Also, it exhibited high MICs of oxytetracycline (64 mg/L), doxycycline (8 mg/L), ampicillin (64 mg/L), amoxicillin (64 mg/L), gentamicin (256 mg/L), streptomycin (128 mg/L), sulfamethoxazole/trimethoprim (513/27 mg/L), and enrofloxacin (8 mg/L).

**FIGURE 1:**
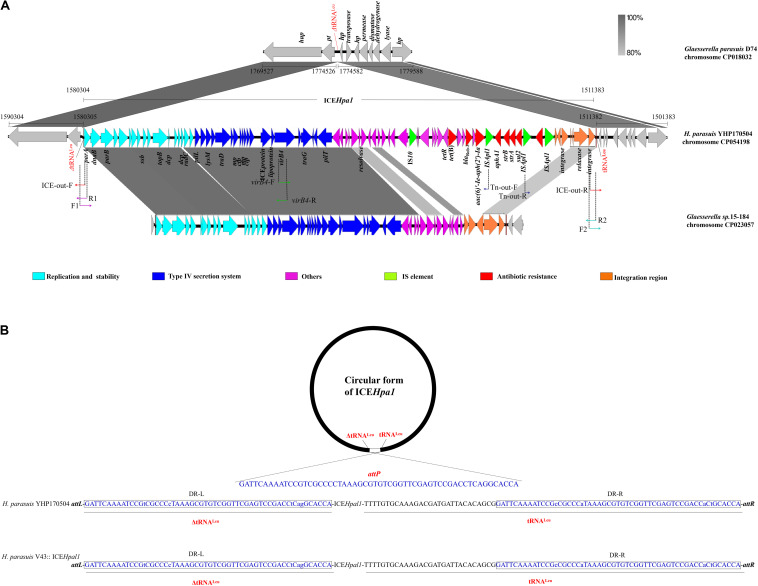
**(A)** Schematic representation of ICE*Hpa1* and its border regions. Genes are presented as broad arrows, with the arrowhead indicating the direction of transcription. Linear comparison of ICE*Hpa1* and its border regions with the homologous region of the genome of *Glaesserella parasuis* (*H. parasuis*) strain D74 (CP018032), and *Glaesserella* sp. (*H. parasuis*) strain 15–184 (CP023057). Thin arrows represent the orientation of each primer and relative positions of the primers along the tested linear sequence. *mp*, gene encoding membrane protein; *ctp*, gene encoding conjugal transfer protein. **(B)** Site-specific integration of ICE*Hpa1* into the tRNA^Leu^ of donor and recipient strain. The sequences of the tRNA^Leu^ are shown in the orientation that matches the orientation of the ICE*Hpa1* sequence. Two tRNA^Leu^ copies (underlined, a truncated copy and an intact copy), the sequences involved in the crossover (*attP* sequence, 56 bp), and the resulting imperfect direct repeats (with lowercase letters indicating the bases that differ) located on the left termini (DR-L, in the closed boxes) and on the right termini (DR-R, in the closed boxes) of the inserted ICE*Hpa1* are also shown.

The chromosome of *H. parasuis* YHP170504 is 2,520,015 bp long with a GC content of 39.64%. Sequence analysis showed that the genome harbors seven resistance genes including the β-lactamase-encoding gene *bla*_Rob–__3_, tetracycline resistance gene *tet*(B), the aminoglycoside resistance genes (*aphA1*, *strA*, and *strB*), aminoglycoside and fluoroquinolone resistance gene [*aac(6)′-Ie-aph(2′)-Ia*], and sulfonamide resistance gene *sul2*. WGS analysis showed that all seven resistance genes were located on a novel integrative conjugative element, designated as ICE*Hpa1* (Figure 1A) according to the nomenclature of ICEs^2^.

ICE*Hpa1* (68,922 bp, corresponding to bases 1,511,383–1,580,304 in GenBank accession number CP054198 or bases 5938–74,859 in GenBank accession number MN844034), a novel ICE with a lower GC content (37.42%), differing from the GC content (39.64%) of entire genome of *H. parasuis* YHP170504, was integrated into the tRNA^Leu^, a common insertion site for ICEs in other species of the family *Pasteurellaceae* (Juhas et al., 2007; Brenner et al., 2012; Eidam et al., 2015; Bossé et al., 2016). WGS analysis showed that the ICE*Hpa1* was flanked by two tRNA^Leu^ copies (a truncated copy and an intact copy, Figure 1A). The presence of an extrachromosomal circular form of ICE*Hpa1* was confirmed in YHP170504 using primers ICE-out-F and ICE-out-R. Sequencing of the closed circular form of ICE*Hpa1* showed that the junction (*attP* sequence) sequence was formed by 56 bp imperfect direct repeats (Figure 1B).

PCR assays and susceptibility testing confirmed the presence and activity of the ICE*Hpa1*-associated resistance genes in the *H. parasuis* V43. The transconjugant *H. parasuis* V43::ICE*Hpal1* showed, in comparison with *H. parasuis* V43, increased MICs of oxytetracycline (from < 0.5 to 32 mg/L), doxycycline (from < 0.5 to 8 mg/L), ampicillin (from 1 to 64 mg/L), amoxicillin (from 1 to 64 mg/L), gentamicin (from < 0.5 to 128 mg/L), streptomycin (from 2 to 128 mg/L), sulfamethoxazole/trimethoprim (from 1.9/0.1 to 513/27 mg/L), and enrofloxacin (from < 0.5 to 4 mg/L). Also, the transconjugant *H. parasuis* V43::ICE*Hpal1* showed the same serovar and ST as *H. parasuis* V43. Although only *traD*, *traG*, *virB4*, *pilT*, and *pilL* genes encoding components of the type IV secretion system were identified, many other conjugal transfer proteins and membrane proteins were present in ICE*Hpa1*, which may also be involved in the conjugal transfer and responsible for the host specificity of the ICE. The ICE*Hpa1* could be transferred to *H. parasuis* V43 at a low frequency of conjugation, with 6.1 × 10^–6^ transconjugants per donor. Our results revealed that ICE*Hpa1* has the self-transmissible capacity to facilitate the dissemination of the resistance genes.

Sequence analysis indicated that the insertion point of ICE*Hpa1* in the transconjugant was located in a tRNA^Leu^. Also, the tRNA^Leu^ locus, in which the ICE*Hpa1* was inserted in the *H. parasuis* V43, showed the same sequence as the one in the *H. parasuis* YHP170504 (Figure 1B). The tRNA^Leu^ into which ICE*Hpa1* was inserted in *H. parasuis* YHP170504 was flanked on one side by a peptide transporter and on the other side by a hypothetical protein. A comparison between the genome of *H. parasuis* YHP170504 and another similar genome of *Glaesserella parasuis* (*H. parasuis*) D74 showed that the genome fragment flanking the left border and right border regions of the ICE*Hpa1* in YHP170504 had high identity to the D74, but a 55-bp truncated tRNA^Leu^ was replaced by the ICE*Hpa1* insertion in YHP170504. Similarly, a peptide transporter and a hypothetical protein were found immediately up- and downstream of the tRNA^Leu^ in *Glaesserella parasuis* D74. The result revealed the strain *Glaesserella parasuis* D74 may serve as a potential recipient for acquiring ICE*Hpa1.*

A total of 81 genes flanked by two tRNA^Leu^ copies were identified within ICE*Hpa1*, of which 17 coded for the putative replication, 20 for type IV secretion system (T4SS), 7 for integration, 4 for transposases of IS elements (an IS*10* and three copies of IS*Apl1*), and 7 for resistance genes including the *bla*_Rob–__3_, *tet*(B), *aphA1*, *strA*, *strB*, *aac(6)′-Ie-aph(2′)-Ia*, and *sul2*. Comparative sequence analysis (Figure 1A) showed that the complete sequence of ICE*Hpa1* shared only 63% identity (the highest rate of match) with the region of *Glaesserella* sp. (*H. parasuis*) 15–184 chromosome, differing clearly from the previous reports about the ICEs from the other *Pasteurellaceae* species (Juhas et al., 2007; Brenner et al., 2012; Eidam et al., 2015; Bossé et al., 2016; Li Y. et al., 2018).

**FIGURE 2:**
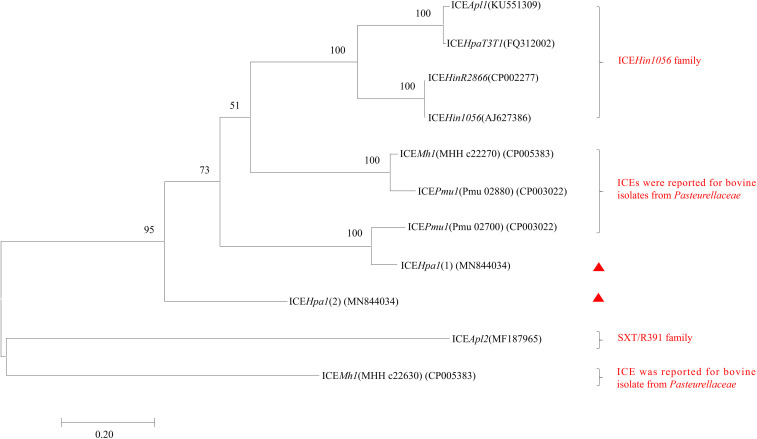
Maximum-likelihood phylogenetic tree based on 11 integrases complete sequences of 8 ICEs from *Pasteurellaceae* by using MEGA7. Two integrases of ICE*Hpa1* are indicated by red triangles. The percentage of trees in which the associated taxa clustered together is shown next to the branches. The tree is drawn to scale, with branch lengths measured in the number of substitutions per site. All positions containing gaps and missing data were eliminated. Bootstrap analysis was performed with 1000 replications. Bar, 0.2 substitution per nucleotide position.

ICE*Hpa1* contains five components (replication and stabilization, T4SS, antimicrobial resistance region, integration and accessory region) (Figure 1A), whose replication and T4SS region shared 98.18% identity with the corresponding region of *Glaesserella* sp. 15–184 chromosome. The accessory region and integration region (including two integrase genes and a relaxase gene) exhibited only partial homology to corresponding region of *Glaesserella* sp. 15–184 chromosome (69% coverage with 96.41% identity and 61% coverage with 85.05% identity, respectively) (Figure 1A). Similar to ICE*Pmu1* in *P. multocida* (Brenner et al., 2012), two integrases were found in ICE*Hpa1*. Identity of 47.35% (93% coverage) was seen when the amino acid sequences of these two integrases were aligned. Comparative sequence analysis revealed that these two integrases are tyrosine recombinases of the Xer family, which are responsible for the integration by site-specific recombination. The integrase 1 in ICE*Hpa1*, belonging to tyrosine recombinase XerD, shared 93.98% (98% coverage) amino acid identity to integrase (Pmu_02700) of ICE*Pmu1* (CP003022). The integrase 2 in ICE*Hpa1*, belonging to tyrosine recombinase XerC, shared 100% (100% coverage) amino acid identity to integrase of *Glaesserella parasuis* strain F9 (KEZ23006.1). However, both of them differed from the integrases reported in ICEs from *Pasteurellaceae* according to the maximum-likelihood tree obtained by using MEGA 7 software (Figure 2). Other experiments are necessary to show which of them or if both are responsible for the integration of ICE*Hpa1.*

**FIGURE 3:**
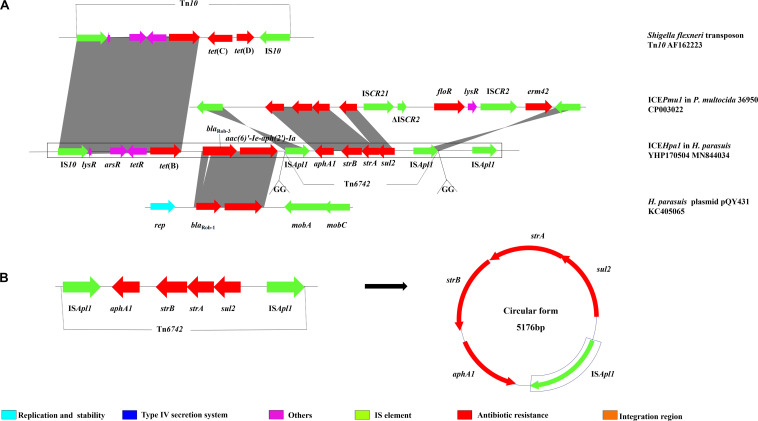
**(A)** Schematic representation the resistance gene region of ICE*Hpa1*. Genes are presented as arrows, with the arrowhead indicating the direction of transcription. Linear comparison of resistance gene region of ICE*Hpa1* with the homologous region of the transposon Tn*10* of *Shigella flexneri* (AF162223), plasmid pQY431 of *H. parasuis* (KC405065), and ICE*Pmu1* of *P. multocida* 36950 (CP003022). **(B)** The circular form of the IS*Apl1-aphA1*-*strB*-*strA-sul2-*IS*Apl1* segment in ICE*Hpa1*.

The multiresistance region (Figure 3A) contains three segments harboring seven different resistance genes. The first segment harboring *tet*(B) was characterized by a truncated transposon Tn*10*, which shows 99% identity with the corresponding region of the Tn*10* transposon. The second segment carries two resistance genes, *bla*_Rob–__3_ and *aac(6)′-Ie-aph(2′)-Ia.* Notably, although this region showed 99% identity with the corresponding region of the *H. parasuis* pQY431 complete sequence, its *bla*_Rob_ gene significantly differed from its counterpart in *H. parasuis* pQY431. Compared with the Bla_Rob_ protein encoded by *bla*_Rob–__1_ from the *H. parasuis* pQY431, two alanine residues [leucine (L) and threonine (T)] were added between positions 16 and 17 in the Bla_Rob_ from ICE*Hpa1.* The Bla_Rob_ from ICE*Hpa1* shared 100% amino acid identity with the Bla_Rob–__3_ from *Moraxella pluranimalium* CCUG 54913 (NG059331). Resistance to β-lactam antibiotics in *H. parasuis* is conferred by two potential mechanisms: mutations in the *ftsI* gene encoding PBP3 and/or production of β-lactamases. WGS analysis showed that no point mutation was found in the *ftsI* gene and no other *bla* gene was detected in *H. parasuis* YHP170504 except *bla*_Rob–__3_, which suggests that the *bla*_Rob–__3_ gene conferred resistance to ampicillin and amoxicillin. To our knowledge, this is the first report of *bla*_Rob–__3_ gene in *Pasteurellaceae* species. The third segment harboring a resistance module *aphA1*-*strB*-*strA-sul2*, which is flanked by two IS*Apl1* elements oriented in the same direction, was regarded as a putative small transposon designated Tn*6742* (Figure 3B). To confirm the excision and cyclization of this structure, PCR was conducted using the primers, Tn-out-F and Tn-out-R. The result showed this structure can be looped out, which indicated IS*Apl1* might accelerate the dissemination of the module *aphA1*-*strB*-*strA-sul2*. IS*Apl1* has been reported to produce a 2 bp direct duplication GG at its integration site (Tegetmeyer et al., 2008). The 2 bp direct duplication GG was detected upstream of the left-hand copy and downstream of the right-hand copy of IS*Apl1*, which suggested that the transposon Tn*6742* was reassembled into the host chromosome via IS*Apl1*-mediated insertion rather than homologous recombination. However, the formation of a similar structure IS*Apl1*-*aphA1*-*strB*-*strA*-*sul2*-IS*CR21-*Δ*ISCR2-floR-ISCR2-erm42-*IS*Apl1* in ICE*Pmu1* from *P. multocida* was proved to insert the chromosome by producing a 2-bp direct duplication GT rather than GG (Brenner et al., 2012). In addition, all the four resistance genes *aphA1*, *strB*, *strA*, and *sul2* oriented in the same direction, which is opposite the direction of the two IS*Apl1*. Compared with that, the four genes and two IS*Apl1*, all oriented in the same direction, were identified in ICE*Pmu1* from *P. multocida* (Brenner et al., 2012). Additional experiments are necessary to show the role of the third IS*Apl1* in the transmission of these resistance genes.

## Conclusion

In summary, a novel ICE was identified from a serovar 8 *H. parasuis* ST288 isolate. To our knowledge, this is the first time a multidrug-resistance ICE has been reported in *H. parasuis*. A total of seven different resistance genes were located on the ICE*Hpa1*, which suggests that the ICE*Hpa1* may act as a reservoir for various resistance genes. Therefore, more research and effective surveillance is needed to monitor the dissemination of multidrug-resistance ICEs.

## Data Availability Statement

The datasets presented in this study can be found in online repositories. The names of the repository/repositories and accession number(s) can be found in the article/supplementary material.

## Ethics Statement

This study was carried out in accordance with the guidelines of Henan Agricultural University Animal Ethics Committee. The owners of the farm animals from which samples were taken gave permission for their animals to be used in this study.

## Author Contributions

G-ZH conceived and designed the experiments. H-RS, X-DC, X-KL, and K-FY produced the data. H-RS, Y-SP, D-DH, HW, LY, and G-ZH analyzed the data. H-RS and G-ZH wrote the manuscript. All authors contributed to the article and approved the submitted version.

## Conflict of Interest

The authors declare that the research was conducted in the absence of any commercial or financial relationships that could be construed as a potential conflict of interest.

## Footnotes

1 http://rast.nmpdr.org

2 https://db-mml.sjtu.edu.cn/ICEberg/
